# Twelve-month mental health service use in six countries of the Americas: A regional report from the World Mental Health Surveys

**DOI:** 10.1017/S2045796019000477

**Published:** 2019-08-27

**Authors:** G. Borges, S. Aguilar-Gaxiola, L. Andrade, C. Benjet, A. Cia, R. C. Kessler, R. Orozco, N. Sampson, J. C. Stagnaro, Y. Torres, Maria Carmen Viana, M. E. Medina-Mora

**Affiliations:** 1 Instituto Nacional de Psiquiatría Ramón de la Fuente Muñiz, Ciudad de México, México; 2 Center for Reducing Health Disparities, UC Davis Health System, Sacramento, California, USA; 3 Núcleo de Epidemiologia Psiquiátrica – LIM 23, Instituto de Psiquiatria Hospital das Clinicas da Faculdade de Medicina da Universidade de São Paulo, São Paulo, Brazil; 4 Anxiety Disorders Center, Buenos Aires, Argentina; 5 Department of Health Care Policy, Harvard Medical School, Boston, Massachusetts, USA; 6 Departamento de Psiquiatría y Salud Mental, Facultad de Medicina, Universidad de Buenos Aires, Buenos Aires, Argentina; 7 Center for Excellence on Research in Mental Health, CES University, Medellin, Colombia; 8 Department of Social Medicine and Post-Graduate Program in Public Health, Psychiatric Epidemiology Research Center (CEPEP), Federal University of Espírito Santo (UFES), Vitória, Brazil

**Keywords:** Epidemiology, mental health, service use, transcultural

## Abstract

**Aims:**

To provide cross-national data for selected countries of the Americas on service utilization for psychiatric and substance use disorders, the distribution of these services among treatment sectors, treatment adequacy and factors associated with mental health treatment and adequacy of treatment.

**Methods:**

Data come from data collected from 6710 adults with 12 month mental disorder surveys across seven surveys in six countries in North (USA), Central (Mexico) and South (Argentina, Brazil, Colombia, Peru) America who were interviewed 2001–2015 as part of the World Health Organization (WHO) World Mental Health (WMH) Surveys. DSM-IV diagnoses were made with the WHO Composite International Diagnostic Interview (CIDI). Interviews also assessed service utilization by the treatment sector, adequacy of treatment received and socio-demographic correlates of treatment.

**Results:**

Little over one in four of respondents with any 12 month DSM-IV/CIDI disorder received any treatment. Although the vast majority (87.1%) of this treatment was minimally adequate, only 35.3% of cases received treatment that met acceptable quality guidelines. Indicators of social-advantage (high education and income) were associated with higher rates of service use and adequacy, but a number of other correlates varied across survey sites.

**Conclusions:**

These results shed light on an enormous public health problem involving under-treatment of common mental disorders, although the problem is most extreme among people with social disadvantage. Promoting services that are more accessible, especially for those with few resources, is urgently needed.

## Introduction

Around the world, mental disorders are very common (Demyttenaere *et al*., 2004), produce a large disease burden (Vos *et al*., 2015; Alonso *et al*., 2018) but are undertreated or receive treatment that does not adhere to evidence-based recommendations (Wang *et al*., 2002). This situation is even worse in low and middle-income countries (Degenhardt *et al*., 2017; Thornicroft *et al*., 2017), where a few resources available are often spent on highly specialised mental health professionals acting in tertiary care settings that tend to privilege severe cases, while general medical professionals in primary care lack training and resources for treating mental disorders (WHO and AIMS 2013).

Qualitative assessments from the Pan-American Health Organization (PAHO) have shown that the organization of mental health services in the region also varies widely (WHO and AIMS 2013; Kohn, 2014). Some countries offer a large range of mental health services based on community mental health care and general physicians, while other countries still rely on psychiatrists in large mental health hospitals that focus mainly on severe mental disorders as their basis of mental health care (Rodríguez, 2007). Quantitative estimates focusing on the state of mental health care in some countries in the region (Brazil, Colombia, Mexico, Peru, the United Sates, Argentina and Medellín-Colombia) have been published since 2005 (Posada-Villa *et al*., 2004; Wang *et al*., 2005, 2007, 2017; Borges *et al*., 2006; Torres de Galvis, 2012; Piazza and Fiestas, 2014; Stagnaro *et al*., 2018), documenting this situation country-by-country. We lack in the Americas a more complete and uniform set of results compared to other regions (Alonso *et al*., 2004) or for worldwide comparisons (Wang *et al*., 2007; Degenhardt *et al*., 2017; Thornicroft *et al*., 2017).

The goal of this report is to provide cross-national data for selected countries of the Americas on mental health service use from a broad list of service providers for mental health and substance use disorders, the distribution of these services among treatment sectors, treatment adequacy and the factors associated with mental health treatment and adequacy of treatment. We additionally present data on comorbidity and disorder severity across countries, which may explain further differences in the rates of service use across the region (Evans-Lacko *et al*., 2018).

## Methods

### Sample

Seven World Health Organization (WHO) WMH surveys were carried out in six countries in the region of the Americas (two surveys in Colombia): two low- and lower-middle-income countries (Colombia-national and Peru), three upper-middle-income countries (Brazil, Colombia-Medellin and Mexico) and two high-income countries (Argentina and the USA) (online Supplementary Table 1S-Annex). One survey was based on a nationally representative household sample (the USA), three (Argentina, Colombia-national and Mexico) on samples representative of urban areas and the remaining three were representative of selected metropolitan areas (Brazil-Sao Paulo, Colombia-Medellin and Peru). In the latter cases, the surveys represented either only one area (São Paulo in Brazil and Medellin in Colombia) or five urban areas (Metropolitan Lima, Huancayo, Iquitos, Arequipa and Chiclayo in Peru). Trained lay interviewers conducted face-to-face interviews with respondents aged **⩾**18 years in all surveys. Respondents were selected using multistage household probability samples. The total sample size was 35 645. The weighted average response rate across all countries was 79.8%. The local human participants' committees approved all surveys. After applying subsampling procedures to reduce respondent burden (Heeringa *et al*., 2008) we focus here on 6710 participants that reported a mental disorder in the last 12 months.

### Measures

The computer-assisted personal interview-version of the WHO World Mental Health (WMH) Survey Initiative-Composite International Diagnostic Interview (CIDI) (Robins *et al*., 1988; Kessler and Üstün, 2004) was administered by a lay interviewer in face-to-face interviews; this fully structured diagnostic interview yielded-DSM-IV diagnoses.

### Disorders

We reported on the 12 month rate of service use for the following categories of mental and substance use disorders: (1) affective disorders: major depressive disorder, dysthymia and bipolar disorder (we used a broad definition that included bipolar I, II and sub-threshold); (2) anxiety disorders: panic disorder, agoraphobia, social phobia, specific phobia, adult separation anxiety disorder, generalised anxiety disorder and posttraumatic stress-disorder; (3) substance use disorders: alcohol and drug abuse and dependence and (4) behavioural disorders: attention deficit/hyperactive disorder and intermittent explosive disorder. We also counted the number of individual disorders (comorbidity) and grouped them as exactly one, exactly two, exactly three and four or more disorders.

### Disorder severity

WMH-CIDI disorders were classified as serious, moderate or mild (Demyttenaere *et al*., 2004; Evans-Lacko *et al*., 2018). The criteria for a serious disorder was the presence of a 12 month bipolar I disorder, substance dependence with a physiological dependence syndrome, a suicide attempt in the past 12 months in conjunction with any other 12 month WMH-CIDI disorder, or if they had at least one 12 month diagnosis and a high level of impairment on the Sheehan Disability Scales (SDS) (Endicott *et al*., 1976; Sheehan *et al*., 1996). Respondents not classified as having a serious disorder were classified as moderate if interference was rated as at least moderate in any SDS domain or if the respondent had substance dependence without a physiological dependence syndrome. The remaining respondents with any 12 month disorder were categorised as mild.

### Treatment sectors

Information about the receipt of 12 month treatment for emotional, alcohol, or drug problems, the type and context of professionals visited, as well as the use of self-help or support groups and hotlines was obtained. Respondents could select as many professionals and treatment options as they used in the previous 12 months. Mental health care in the 12 months before the survey was divided into the following five sectors: (1) psychiatrists; (2) other mental health specialists, consisting of psychologists, counselors, psychotherapists, mental health nurses and social workers in a mental health specialty setting; and (3) general medical practitioners, consisting of family physicians, general practitioners and other medical doctors, such as cardiologists, or gynecologists (for women) and urologists (for men), nurses, occupational therapists, or other health care professionals; (4) human services, including outpatient treatment with a religious or spiritual advisor or a social worker or counselor in any setting other than a specialty mental health setting, or a religious or spiritual-advisor, such as a minister, priest, or rabbi and (5) complementary-alternative medicine included internet use, self-help groups, any other healer, such as an herbalist, a chiropractor, or a spiritualist and other alternative therapy. We further grouped psychiatrists and other mental health specialist as *any mental health specialist*; and psychiatrists, mental health specialists and general medical care practitioners under *any health care*.

### Minimally adequate mental health care

We used three definitions of treatment adequacy during the prior 12 months. First, we defined follow-up care (a ‘very light’ definition of treatment) as at least two visits in any service sector in the past 12 months or being currently in treatment (Wang *et al*., 2007). Second, with available evidence-based treatment guidelines for primary-care (Panel, 1993) and specialty mental health providers (American Psychiatric Association, 1994, 1997, 1998, 2000; Lehman and Steinwachs, 1998), we defined minimally adequate treatment (a ‘light’ definition which is used as the main one in this paper) as receiving: (1) minimally adequate psychotherapy, consisting of four or more outpatient visits to any provider (Sturm and Wells, 1995; Young *et al*., 2001); (2) minimally adequate pharmacotherapy, consisting of two or more outpatient visits to any provider and treatment with any medication for any length of time (National Committee for Quality Assurance, 1999) and (3) reporting still being ‘in treatment’ at the time of the interview. Although this definition is broader than the next one (Kessler *et al*., 2003), it allowed us to obtain conservative estimates of minimally adequate treatment across sectors. Third, a ‘stringent’ definition of minimally adequate treatment was used, in which we required: (1) eight or more visits to any service sector for psychotherapy or (2) four or more visits to any service sector and 30 or more days taking any medication for pharmacotherapy.

### Socio-demographic predictor variables

Socio-demographic variables included age (18–34, 35–49, 50–64 and 65+ years), sex and marital status (married/cohabitating, previously married, never married). Completed years of education (low, low average, high average and high) and family income (low, low-average, high-average and high) were defined based on country-specific distributions, as detailed in other work from our group (Evans-Lacko *et al*., 2018).

### Analyses

The data was weighted to adjust for differential probabilities of selection and nonresponse. Estimates of standard errors for proportions were obtained by the Taylor series-linearization method with SAS' survey analysis procedures (Research Triangle Park, 2002). Logistic regression-analysis (Hosmer and Lemeshow, 2000) was performed to study socio-demographic correlates. Two sets of parallel analyses were performed, the first ones for receiving treatment and the second one for receiving minimally adequate treatment among those who received any treatment. Estimates of standard errors of odds ratios and their corresponding standard errors from logistic regression coefficients were also obtained with SAS software, and 95% confidence intervals were adjusted for design effects. Statistical significance was evaluated with two-sided design-based tests with 0.05 level of significance.

All models included controls for survey and mental disorders. Inspection of Akaike's Information Criterion (AIC) (Burnham and Anderson, 2002) in preliminary models favoured including controls for group of disorders (any anxiety, any mood, any substance and any behavioural) over individual disorders. Models examined between-country variation in associations with socio-demographic variables by including in the model all predictor-by-survey interactions using a dummy coding scheme that kept the product of all country-specific ORs equal to 1. This method allowed us to detect significant between-country variation with respect to the overall effect, by evaluating the statistical significance of deviation of within-survey coefficients from the median 1.0 value (Mortier *et al*., 2018). The reported survey-specific ORs show to what extent the survey-specific effect deviates from the overall effect. For example, if the reported OR for females (versus males) in the U.S. is 1.5, then it would be necessary to multiply it by the reported overall effect OR  =  1.2 to obtain the survey-specific effect in the U.S. (i.e. OR  =  1.8).

## Results

### Prevalence of 12 month mental health service use

Overall, of the respondents with any disorder 27.6% reported any service use (Table 1). The prevalence across sites for any service use among those with any disorder varied from 13.1% in Colombia-national to 39.7% in the USA. The highest rate of any service use for all sites and overall was for any mood disorder (40.4%; range 21.7% in Colombia-national to 56.1% in the United States) and, overall, the lowest among those with substance use disorders (24.6%; range 8.0% in Colombia-national to 39.6% in the United States) in all sites but in Mexico and the United States where those with an externalised disorders ranked the lowest. Most of the treatments were delivered by the health care sector (24.4% for any disorder), ranking first place in all sites. Within the health care sector, any mental health specialist had the largest share of treatment use (15.6% for any disorder, overall) in all sites except in the United States, where a general medical practitioner was the resource most used among those with any disorder. About one in every three respondents with a mood disorder in the United States used a general medical practitioner for treatment. The psychiatrist was the resource least used within the health care sector overall in all sites (8.5% overall for any disorder, range 3.4% in Colombia-national to 11.6% in the United States) except in Brazil where other mental health specialist ranked the lowest.

**Table 1.** Twelve month treatment of mental disorders, overall and within separate service sectors among WMH respondents with 12 month DSM-IV/CIDI disorders, by survey in the PAHO region (*n*  =  6710)

		No. of respondents with disorder	Psychiatrist		Other mental health specialist		Any mental health specialist		General medical		Any health care		Human services		CAM		Any service use	
Group of disorders	Survey	Unweighted *n*	Unweighted *n*	% (s.e.)	Unweighted *n*	% (s.e.)	Unweighted *n*	% (s.e.)	Unweighted *n*	% (s.e.)	Unweighted *n*	% (s.e.)	Unweighted *n*	% (s.e.)	Unweighted *n*	% (s.e.)	Unweighted *n*	% (s.e.)
Anxiety disorders																		
	Argentina	363	44	8.7 (1.9)	61	16.1 (2.8)	82	19.2 (2.8)	56	14.1 (2.2)	119	29.6 (3.3)	7	1.0 (0.3)	7	1.2 (0.6)	124	30.6 (3.3)
	Brazil	797	109	11.3 (1.2)	65	7.7 (0.9)	143	15.6 (1.2)	83	8.1 (1.3)	206	21.1 (1.6)	27	3.1 (0.7)	30	4.0 (0.9)	227	23.5 (1.6)
	Colombia	584	20	2.7 (0.9)	37	4.6 (1.1)	51	6.9 (1.4)	36	5.9 (1.1)	83	12.4 (1.8)	4	–	5	0.7 (0.4)	88	13.2 (1.9)
	Medellin Colombia	386	27	5.8 (1.2)	29	6.8 (1.5)	48	10.8 (1.7)	34	8.9 (1.9)	76	18.2 (2.3)	6	1.8 (0.8)	8	0.8 (0.3)	85	19.6 (2.4)
	Mexico	447	17	3.5 (1.0)	30	6.6 (1.3)	42	9.0 (1.7)	34	5.8 (1.3)	71	13.8 (2.2)	4	–	11	3.3 (1.3)	79	16.0 (2.5)
	Peru	249	14	4.4 (1.1)	13	4.3 (1.5)	26	8.5 (2.1)	15	5.6 (1.8)	41	14.1 (2.9)	9	3.7 (1.1)	9	2.8 (0.9)	53	18.8 (3.6)
	United States	1744	234	13.4 (1.0)	294	16.4 (1.0)	402	22.3 (1.2)	421	23.9 (1.0)	667	37.3 (1.3)	145	8.2 (0.8)	134	7.2 (0.7)	765	42.6 (1.1)
	Overall	4570	465	9.5 (0.5)	529	11.1 (0.5)	794	16.2 (0.7)	679	14.3 (0.6)	1263	26.0 (0.8)	202	4.5 (0.4)	204	4.3 (0.4)	1421	29.3 (0.8)
Mood disorders																		
	Argentina	261	43	11.2 (2.3)	54	21.2 (3.2)	78	26.9 (3.2)	26	9.4 (2.0)	94	32.6 (4.2)	7	2.0 (0.9)	6	2.5 (1.2)	101	35.5 (4.2)
	Brazil	570	104	16.7 (1.9)	60	10.5 (1.7)	134	22.6 (2.4)	77	14.9 (1.5)	188	32.1 (1.9)	32	6.6 (1.6)	27	4.5 (1.0)	214	36.6 (2.2)
	Colombia	309	20	7.7 (2.3)	26	7.2 (1.9)	42	14.2 (3.0)	29	7.6 (1.8)	65	20.5 (3.5)	5	–	2	–	69	21.7 (3.5)
	Medellin Colombia	177	17	8.4 (2.3)	23	12.8 (2.8)	32	17.1 (3.1)	13	7.2 (2.1)	43	22.9 (3.7)	6	–	5	1.3 (0.6)	49	25.5 (3.8)
	Mexico	298	12	4.3 (1.6)	27	8.3 (1.6)	38	12.3 (2.2)	36	9.4 (1.5)	70	20.5 (2.4)	6	1.1 (0.5)	12	4.3 (1.5)	83	24.2 (2.6)
	Peru	131	9	6.3 (2.2)	16	11.4 (3.4)	22	15.8 (3.8)	13	10.4 (2.6)	34	25.4 (3.6)	4	–	7	4.9 (1.8)	39	28.8 (4.1)
	United States	922	189	20.5 (1.3)	218	23.8 (1.5)	299	32.4 (1.3)	290	32.8 (1.9)	461	50.6 (2.0)	104	10.8 (1.2)	93	9.9 (1.3)	516	56.1 (1.9)
	Overall	2668	394	14.8 (0.8)	424	16.1 (0.9)	645	24.3 (1.0)	484	19.4 (1.1)	955	36.2 (1.3)	164	6.6 (0.7)	152	5.8 (0.6)	1071	40.4 (1.3)
Substance use disorders																		
	Argentina	73	11	7.7 (3.4)	7	10.6 (3.7)	15	14.8 (4.3)	6	–	20	20.6 (5.7)	2	–	2	–	21	22.1 (5.8)
	Brazil	164	18	9.4 (2.7)	16	10.2 (3.6)	27	15.9 (4.3)	10	5.1 (1.9)	30	17.5 (4.4)	3	–	8	5.6 (2.3)	32	19.4 (4.6)
	Colombia	90	6	3.4 (1.7)	4	–	9	4.9 (2.0)	2	–	11	7.4 (2.8)	0	0 (0)	2	–	12	8.0 (3.1)
	Medellin Colombia	85	5	3.9 (2.0)	7	7.9 (3.3)	10	9.7 (3.5)	2	–	12	10.5 (3.5)	1	–	5	–	15	11.8 (3.8)
	Mexico	80	5	–	8	6.8 (3.0)	12	14.0 (4.8)	4	–	15	16.2 (4.7)	0	0 (0)	5	–	19	18.6 (4.8)
	Peru	50	3	–	2	–	5	9.1 (4.5)	0	0 (0)	5	9.1 (4.5)	0	0 (0)	2	–	7	11.7 (5.1)
	United States	314	46	13.2 (1.4)	68	20.6 (2.3)	85	25.6 (1.9)	67	20.0 (2.1)	118	35.8 (2.7)	21	7.0 (1.8)	29	7.6 (1.7)	132	39.6 (2.7)
	Overall	856	94	9.0 (0.9)	112	12.4 (1.4)	163	17.0 (1.4)	91	9.5 (1.2)	211	22.1 (1.8)	27	3.2 (0.8)	53	5.0 (0.8)	238	24.6 (1.9)
Externalised disorders																		
	Argentina	41	7	12.9 (6.0)	5	–	9	20.8 (9.6)	6	11.1 (5.5)	13	27.4 (8.4)	5	9.9 (4.6)	1	–	15	30.6 (8.8)
	Brazil	217	19	5.8 (1.7)	18	8.6 (2.4)	28	10.9 (2.6)	22	8.3 (1.9)	43	16.7 (3.5)	6	2.1 (0.9)	11	5.2 (1.9)	50	20.5 (3.7)
	Colombia	147	11	5.3 (1.7)	9	4.8 (1.9)	18	9.8 (2.2)	11	5.2 (2.0)	26	14.0 (3.1)	2	–	1	–	28	14.5 (3.1)
	Medellin Colombia	59	5	–	3	–	7	8.8 (3.6)	3	–	10	13.5 (4.9)	0	0 (0)	0	0 (0)	10	13.5 (4.9)
	Mexico	88	3	–	7	8.0 (4.0)	10	10.1 (4.2)	7	5.2 (2.3)	16	14.1 (4.4)	0	0 (0)	3	–	18	15.0 (4.5)
	Peru	81	3	–	16	21.8 (6.2)	17	22.7 (6.4)	1	–	18	23.5 (6.6)	3	–	4	–	20	25.4 (6.6)
	United States	659	75	11.2 (1.4)	106	15.5 (1.6)	138	20.4 (1.9)	128	18.8 (2.2)	214	31.6 (1.9)	52	8.8 (1.9)	42	5.7 (1.0)	253	37.6 (2.1)
	Overall	1292	123	8.3 (0.9)	164	12.5 (1.1)	227	16.6 (1.3)	178	12.7 (1.3)	340	24.5 (1.4)	68	5.5 (1.1)	62	4.3 (0.6)	394	28.7 (1.5)
Any disorder																		
	Argentina	568	66	7.9 (1.5)	87	14.3 (2.4)	124	18.0 (2.5)	71	11.1 (1.8)	171	26.1 (3.2)	14	1.6 (0.5)	10	1.6 (0.4)	182	27.7 (3.2)
	Brazil	1248	153	10.1 (0.9)	99	7.7 (0.7)	206	14.8 (1.0)	124	8.3 (0.8)	296	20.3 (1.2)	43	3.2 (0.7)	42	3.3 (0.6)	332	22.9 (0.9)
	Colombia	847	32	3.4 (0.8)	52	4.5 (0.8)	76	7.3 (1.1)	54	5.5 (0.9)	123	12.4 (1.5)	8	–	5	0.5 (0.2)	131	13.1 (1.5)
	Medellin Colombia	530	36	5.2 (0.9)	47	8.1 (1.4)	70	11.3 (1.5)	39	6.9 (1.4)	103	17.2 (1.9)	8	1.4 (0.6)	12	1.6 (0.6)	113	18.4 (2.0)
	Mexico	695	27	4.0 (0.8)	52	7.0 (1.2)	74	10.3 (1.5)	58	5.9 (1.0)	124	15.2 (1.6)	7	0.5 (0.2)	20	2.8 (0.9)	142	17.5 (1.7)
	Peru	391	23	4.4 (0.7)	29	7.8 (2.1)	49	11.7 (2.2)	21	4.8 (1.3)	69	16.3 (2.7)	11	3.1 (1.1)	14	2.7 (0.8)	83	19.5 (3.1)
	United States	2431	296	11.6 (0.7)	391	15.5 (0.8)	532	20.8 (0.9)	547	21.8 (0.8)	880	34.7 (1.0)	192	7.8 (0.8)	172	6.5 (0.6)	1008	39.7 (0.9)
	Overall	6710	633	8.5 (0.4)	757	10.8 (0.5)	1131	15.6 (0.5)	914	12.9 (0.5)	1766	24.4 (0.6)	283	4.2 (0.4)	275	3.9 (0.3)	1991	27.6 (0.7)

WMH, World Mental Health; CIDI, Composite International Diagnostic Interview; s.e., standard error.

–– Percentage less than twice the s.e. or sample size < 30

Analyses performed on the part II sample.

Anxiety disorders: panic disorder and/or agoraphobia, specific phobia, social phobia, generalised anxiety disorder, adult separation anxiety disorder and PTSD. Mood disorders: major depressive disorder/dysthymia and bipolar broad. Substance use disorders: alcohol and drug abuse/dependence. Externalised disorders: attention-deficit/hyperactivity disorder and intermittent explosive disorder.

Intermittent explosive disorder was not assessed in Mexico and Medellin and was coded as zero.

Imputed variables for alcohol and drug dependence were used for Colombia, Mexico, Peru and the U.S.

Lifetime ADHD was used in all countries and was coded as zero for those with age > 45 in Colombia, Mexico, Peru and the U.S.

### Number of disorders and severity of mental disorder

Overall and across study sites, there was a clear trend for a higher prevalence of service use with higher number of disorders (except in Colombia-national and Medellin-Colombia) and greater severity of the disorders (except in Argentina). For example, 20.3% of those with only one disorder reported any service use and 50.6% of those with four+ disorders (Table 2). Overall, only 18.2% of those with a mild disorder used any service, but as many as 40.8% for those with a severe disorder. Focusing on those with a higher need, about one in every two respondents with four+ disorders received any health care services in Argentina and about two in every three in the United States, but only one in every six received the same services in Colombia-national and in Medellin-Colombia. Slightly lower percentages were seen for those with a severe mental disorder and any health care use.

**Table 2.** Twelve month treatment of mental disorders, overall and within separate service sectors among WMH respondents with 12 month DSM-IV/CIDI disorders, by number of disorders and severity, by survey in the PAHO region (*n*  =  6710).

		Number of disorders/severity	No. of respondents with disorder	Psychiatrist		Other mental health specialist		Any mental health specialist		General medical		Any health care		Human services		CAM		Any service use	
Variable	Survey		Unweighted *n*	Unweighted *n*	% (s.e.)	Unweighted *n*	% (s.e.)	Unweighted *n*	% (s.e.)	Unweighted *n*	% (s.e.)	Unweighted *n*	% (s.e.)	Unweighted *n*	% (s.e.)	Unweighted *n*	% (s.e.)	Unweighted *n*	% (s.e.)
Number of disorders																			
	Argentina	1	376	29	4.4 (1.0)	46	9.7 (2.1)	64	12.2 (2.4)	46	10.3 (2.2)	96	20.7 (3.2)	7	0.8 (0.4)	2	–	100	21.2 (3.2)
	Argentina	2	112	16	13.8 (5.1)	21	26.5 (5.8)	31	32.0 (5.6)	15	12.5 (4.2)	38	36.3 (5.7)	4	–	5	6.1 (1.9)	42	40.6 (6.1)
	Argentina	3	45	7	13.7 (4.9)	8	18.7 (5.0)	11	25.0 (5.5)	4	–	15	32.7 (6.4)	3	–	0	0 (0)	18	40.0 (7.8)
	Argentina	4+	35	14	26.8 (10.0)	12	28.7 (12.6)	18	39.0 (13.6)	6	21.5 (8.6)	22	55.5 (10.5)	0	0 (0)	3	–	22	55.5 (10.5)
	Brazil	1	727	60	6.4 (1.1)	41	5.3 (0.6)	86	10.0 (1.1)	53	5.4 (0.8)	127	13.8 (1.5)	23	2.6 (0.7)	15	1.8 (0.8)	148	16.0 (1.5)
	Brazil	2	265	39	13.8 (2.8)	23	10.2 (2.6)	47	19.1 (2.4)	28	11.0 (2.5)	68	26.3 (2.5)	11	5.4 (2.2)	8	4.6 (2.2)	74	29.2 (2.8)
	Brazil	3	144	25	14.8 (2.9)	16	10.5 (2.6)	34	20.5 (2.8)	26	16.8 (3.1)	53	32.6 (3.8)	3	–	6	–	57	35.6 (4.5)
	Brazil	4+	112	29	25.9 (5.6)	19	17.9 (3.9)	39	35.8 (5.0)	17	13.2 (3.3)	48	42.1 (5.0)	6	5.2 (2.2)	13	12.7 (3.8)	53	47.3 (6.4)
	Colombia	1	534	12	2.0 (0.8)	25	2.9 (0.8)	35	4.8 (0.9)	26	4.5 (1.0)	59	8.9 (1.4)	4	–	1	–	62	9.3 (1.4)
	Colombia	2	193	10	6.5 (2.8)	18	7.0 (1.7)	26	12.8 (3.0)	17	7.0 (1.9)	39	18.3 (3.2)	2	–	2	–	41	19.3 (3.6)
	Colombia	3	62	5	4.4 (2.2)	5	13.3 (6.4)	8	15.0 (6.7)	5	–	13	22.1 (7.3)	2	–	1	–	16	26.8 (7.3)
	Colombia	4+	58	5	−-	4	−-	7	7.8 (3.7)	6	8.9 (4.4)	12	16.5 (5.6)	0	0 (0)	1	−-	12	16.5 (5.6)
	Medellin Colombia	1	302	16	3.5 (1.0)	29	9.1 (1.9)	39	11.3 (2.0)	19	6.5 (2.0)	54	16.6 (2.7)	3	–	5	–	56	17.2 (2.7)
	Medellin Colombia	2	118	8	7.1 (2.6)	12	9.4 (2.9)	17	13.2 (3.4)	10	5.8 (2.2)	26	17.7 (4.1)	3	–	3	–	28	18.9 (4.2)
	Medellin Colombia	3	58	7	7.8 (3.4)	2	–	7	7.8 (3.4)	6	12.4 (5.3)	13	20.2 (6.1)	2	–	1	–	16	24.2 (6.5)
	Medellin Colombia	4+	52	5	8.4 (3.9)	4	–	7	11.5 (4.4)	4	–	10	15.9 (4.8)	0	0 (0)	3	–	13	17.3 (4.9)
	Mexico	1	430	9	1.6 (0.5)	29	6.9 (1.7)	37	8.2 (1.8)	33	5.7 (1.1)	66	13.1 (1.9)	4	–	10	2.5 (1.0)	76	15.4 (2.0)
	Mexico	2	158	11	9.8 (3.5)	12	5.8 (1.9)	20	14.0 (3.8)	15	5.6 (1.6)	34	19.2 (4.2)	3	–	5	–	38	20.8 (4.4)
	Mexico	3	75	5	–	7	8.0 (3.2)	11	11.5 (3.9)	6	–	16	16.1 (4.8)	0	0 (0)	3	–	18	18.0 (4.8)
	Mexico	4+	32	2	–	4	–	6	23.1 (9.0)	4	–	8	26.4 (9.1)	0	0 (0)	2	–	10	33.5 (8.8)
	Peru	1	259	14	3.9 (1.1)	15	6.5 (2.4)	28	10.0 (2.2)	12	4.3 (1.4)	39	14.1 (2.7)	6	2.3 (0.9)	6	1.5 (0.7)	46	16.6 (2.8)
	Peru	2	91	6	6.0 (2.3)	7	9.4 (3.2)	12	14.5 (3.9)	6	5.1 (2.2)	18	19.6 (5.3)	2	–	4	3.5 (1.7)	21	21.9 (5.5)
	Peru	3	30	2	–	5	–	7	21.0 (7.1)	2	–	9	28.5 (5.2)	2	–	3	–	12	39.4 (11.2)
	Peru	4+	11	1	–	2	–	2	–	1	–	3	–	1	–	1	–	4	–
	United States	1	1215	80	5.6 (0.7)	141	10.7 (1.0)	184	13.6 (1.1)	212	16.9 (1.2)	338	26.3 (1.6)	70	5.2 (0.7)	57	3.9 (0.6)	388	29.8 (1.6)
	United States	2	583	76	13.0 (1.5)	90	15.6 (1.8)	133	22.8 (2.1)	143	24.7 (1.9)	229	38.7 (2.3)	57	10.6 (1.8)	40	6.9 (1.0)	264	44.9 (2.1)
	United States	3	312	54	18.0 (2.9)	66	19.5 (3.1)	88	27.1 (3.4)	85	24.6 (2.3)	135	40.6 (3.6)	23	6.9 (1.5)	29	9.6 (1.7)	153	47.2 (3.9)
	United States	4+	321	86	27.6 (2.4)	94	31.4 (2.6)	127	40.8 (2.6)	107	34.3 (2.7)	178	56.6 (2.7)	42	13.7 (2.1)	46	13.7 (2.1)	203	64.1 (2.5)
	Overall	1	3843	220	4.6 (0.4)	326	7.7 (0.5)	473	10.7 (0.6)	401	9.6 (0.6)	779	18.1 (0.8)	117	2.7 (0.3)	96	2.2 (0.3)	876	20.3 (0.8)
	Overall	2	1520	166	11.4 (1.0)	183	12.6 (1.1)	286	19.7 (1.2)	234	15.2 (1.2)	452	29.7 (1.4)	82	6.4 (1.0)	67	4.9 (0.6)	508	33.5 (1.5)
	Overall	3	726	105	13.5 (1.6)	109	14.5 (1.7)	166	21.5 (1.9)	134	17.4 (1.4)	254	32.9 (2.1)	35	4.7 (0.9)	43	5.8 (1.0)	290	38.1 (2.4)
	Overall	4+	621	142	22.5 (1.9)	139	23.1 (1.9)	206	33.0 (2.1)	145	24.2 (2.0)	281	45.1 (2.3)	49	8.6 (1.2)	69	10.5 (1.4)	317	50.6 (2.4)
Severity																			
	Argentina	Severe	152	31	14.0 (3.4)	29	15.1 (3.9)	47	21.5 (4.0)	20	10.8 (1.9)	57	27.8 (3.6)	6	2.8 (1.3)	4	–	61	30.2 (3.7)
	Argentina	Moderate	218	25	8.9 (2.3)	39	20.0 (4.0)	51	24.0 (3.9)	26	10.4 (2.1)	70	31.8 (4.8)	2	–	4	–	72	33.0 (4.8)
	Argentina	Mild	198	10	2.8 (1.2)	19	8.3 (2.3)	26	10.0 (2.9)	25	11.8 (3.5)	44	19.4 (4.7)	6	–	2	–	49	20.9 (4.8)
	Brazil	Severe	461	92	18.0 (2.1)	56	12.6 (1.6)	118	24.3 (2.2)	58	11.8 (2.0)	156	31.2 (2.2)	25	5.8 (1.3)	27	6.7 (1.3)	170	34.4 (2.2)
	Brazil	Moderate	403	36	8.0 (1.7)	29	7.3 (1.4)	53	12.8 (2.2)	41	7.3 (1.2)	84	17.5 (2.2)	10	–	10	–	97	21.1 (2.9)
	Brazil	Mild	384	25	4.2 (0.9)	14	3.1 (0.9)	35	6.8 (1.1)	25	5.8 (1.4)	56	11.9 (1.9)	8	0.6 (0.3)	5	–	65	12.9 (2.0)
	Colombia	Severe	191	19	11.0 (3.2)	18	8.4 (2.7)	30	17.4 (4.2)	23	9.3 (2.4)	49	25.6 (4.6)	4	–	3	–	54	27.7 (4.8)
	Colombia	Moderate	338	8	1.2 (0.5)	21	3.7 (0.9)	28	4.9 (1.0)	21	5.9 (1.6)	46	10.2 (2.0)	2	–	2	–	47	10.3 (2.0)
	Colombia	Mild	318	5	–	13	2.7 (0.8)	18	3.6 (0.9)	10	2.6 (0.9)	28	6.2 (1.2)	2	–	0	0 (0)	30	6.7 (1.3)
	Medellin Colombia	Severe	157	23	10.9 (2.6)	20	11.0 (2.9)	34	16.9 (3.3)	13	4.8 (1.5)	43	19.9 (3.4)	5	–	4	–	48	22.0 (3.5)
	Medellin Colombia	Moderate	193	9	3.7 (1.3)	11	5.8 (2.0)	17	8.2 (2.3)	16	9.6 (3.1)	33	17.8 (3.6)	1	–	5	1.2 (0.5)	37	18.7 (3.6)
	Medellin Colombia	Mild	180	4	–	16	8.0 (2.4)	19	9.9 (2.5)	10	5.9 (2.1)	27	14.2 (3.0)	2	–	3	–	28	14.8 (3.2)
	Mexico	Severe	179	9	7.7 (3.1)	18	8.6 (2.4)	26	15.5 (3.5)	21	8.2 (2.1)	44	22.2 (4.0)	1	–	8	3.4 (1.3)	52	25.8 (4.3)
	Mexico	Moderate	258	12	4.2 (1.6)	20	7.2 (1.9)	30	10.6 (2.3)	20	6.1 (1.4)	48	16.2 (2.7)	3	–	7	3.1 (1.5)	53	17.9 (2.9)
	Mexico	Mild	258	6	1.7 (0.8)	14	5.9 (1.8)	18	7.2 (1.9)	17	4.4 (1.3)	32	10.5 (2.2)	3	–	5	–	37	12.6 (2.3)
	Peru	Severe	79	13	14.1 (3.7)	8	8.9 (3.4)	19	21.4 (5.2)	6	–	24	27.8 (5.2)	3	–	5	–	28	32.8 (6.5)
	Peru	Moderate	166	4	–	11	6.8 (2.8)	14	8.0 (3.0)	10	5.3 (1.5)	24	13.3 (3.8)	5	3.6 (1.5)	7	3.2 (1.1)	32	18.1 (4.6)
	Peru	Mild	146	6	3.0 (1.3)	10	8.4 (3.5)	16	11.4 (3.5)	5	3.1 (1.5)	21	14.5 (3.8)	3	–	2	–	23	15.3 (4.0)
	United States	Severe	633	166	26.5 (1.9)	179	28.6 (1.9)	250	39.4 (2.1)	204	32.2 (1.8)	348	54.2 (2.6)	76	12.0 (1.4)	75	11.4 (1.3)	385	59.7 (2.4)
	United States	Moderate	963	89	9.0 (1.2)	139	13.7 (1.3)	183	18.0 (1.4)	221	22.5 (1.4)	336	34.1 (1.3)	78	7.6 (0.7)	65	6.2 (1.1)	395	40.0 (1.3)
	United States	Mild	835	41	4.3 (0.8)	73	8.5 (1.1)	99	11.2 (1.2)	122	14.1 (1.3)	196	22.2 (1.7)	38	5.1 (1.3)	32	3.6 (0.7)	228	26.1 (1.8)
	Overall	Severe	1852	353	18.5 (1.0)	328	17.5 (1.0)	524	27.4 (1.2)	345	17.8 (1.0)	721	36.9 (1.4)	120	6.8 (0.7)	126	6.8 (0.6)	798	40.8 (1.3)
	Overall	Moderate	2539	183	6.6 (0.6)	270	10.1 (0.8)	376	13.8 (0.9)	355	13.4 (0.8)	641	23.8 (1.0)	101	4.0 (0.5)	100	3.6 (0.5)	733	27.5 (1.1)
	Overall	Mild	2319	97	3.3 (0.4)	159	6.5 (0.6)	231	8.9 (0.7)	214	8.8 (0.7)	404	16.0 (1.0)	62	2.6 (0.6)	49	2.0 (0.4)	460	18.2 (1.0)

WMH, World Mental Health; CIDI, Composite International Diagnostic Interview; s.e., standard error

– Percentage less than twice the s.e. or sample size < 30

Analyses performed on the part II sample.

Anxiety disorders: panic disorder and/or agoraphobia, specific phobia, social phobia, generalised anxiety disorder, adult separation anxiety disorder and PTSD. Mood disorders: major depressive disorder/dysthymia and bipolar broad. Substance use disorders: alcohol and drug abuse/dependence. Externalised disorders: attention-deficit/hyperactivity disorder and intermittent explosive disorder.

Intermittent explosive disorder was not assessed in Mexico and Medellin and was coded as zero.

Imputed variables for alcohol and drug dependence were used for Colombia, Mexico, Peru and the U.S.

Lifetime ADHD was used in all countries and was coded as zero for those with age > 45 in Colombia, Mexico, Peru and the U.S.

### Minimally adequate treatment

Overall, among those with a 12 month disorder who received any 12 month treatment, 87.1% (range 71.4% in Peru to 89.4% in the United States) of those received follow-up treatment (the ‘very light’ definition of adequacy) (Table 3), and the greatest proportion of follow-up was observed among those with a substance use disorder and the lowest for those with a externalised disorder. The overall prevalence of adequate treatment was 72.8% (range 51.4% in Peru to 77.4% in the United States), but it was only 35.3% when using the stringent definition of adequacy (range 12.4% in Peru to 42.9% in Argentina). Overall, when a ‘stringent’ definition was used, services provided by psychiatrists had greater treatment adequacy than those provided by general medical professionals (19.2 and 15.6%, respectively), but lower than other mental health specialists for which the greatest treatment adequacy was found (23.7%) (online Supplementary Table 2S-Annex).

**Table 3.** Adequacy of treatment of mental disorders among WMH respondents with 12 month DSM-IV/CIDI disorders with any service use, by survey in the PAHO region (*n*  =  1991)

	No. of respondents with disorder	Minimally adequate treatment (‘stringent' definition)			Minimally adequate treatment (‘light' definition)		Follow-up treatment (‘very light' definition)	
Group of disorders	Survey	Unweighted *n*	Unweighted *n*	% (s.e.)	Unweighted *n*	% (s.e.)	Unweighted *n*	% (s.e.)
Anxiety disorders								
	Argentina	124	46	41.6 (5.1)	85	70.5 (5.4)	106	85.1 (3.5)
	Brazil	227	96	46.1 (4.1)	176	78.8 (3.6)	199	89.3 (2.5)
	Colombia	88	15	24.4 (7.0)	47	59.3 (6.2)	69	83.3 (4.9)
	Medellin Colombia	85	19	22.0 (5.9)	59	61.2 (7.2)	73	80.8 (6.2)
	Mexico	79	15	20.3 (5.6)	46	57.2 (6.5)	61	83.1 (3.8)
	Peru	53	3	–	25	47.8 (6.6)	37	70.3 (6.4)
	United States	765	297	38.1 (2.2)	607	78.0 (2.3)	698	89.5 (2.0)
	Overall	1421	491	36.2 (1.6)	1045	73.8 (1.6)	1243	87.4 (1.3)
Mood disorders								
	Argentina	101	43	44.6 (5.6)	75	73.0 (5.2)	88	87.8 (3.9)
	Brazil	214	88	42.3 (5.2)	167	80.0 (4.2)	188	89.8 (3.0)
	Colombia	69	10	18.8 (7.7)	40	64.1 (7.3)	55	83.9 (5.5)
	Medellin Colombia	49	14	28.5 (7.7)	37	74.8 (7.7)	43	89.4 (5.8)
	Mexico	83	18	23.5 (4.5)	48	57.2 (6.2)	62	81.5 (4.6)
	Peru	39	4	–	23	55.8 (7.7)	31	80.5 (6.5)
	United States	516	243	48.1 (2.2)	415	79.3 (2.4)	471	89.8 (2.1)
	Overall	1071	420	41.7 (1.8)	805	76.1 (1.7)	938	88.6 (1.4)
Substance use disorders								
	Argentina	21	5	–	16	–	17	–
	Brazil	32	10	30.2 (9.5)	20	64.9 (9.0)	28	90.9 (4.8)
	Colombia	12	3	–	8	–	12	–
	Medellin Colombia	15	4	–	12	–	12	–
	Mexico	19	2	–	9	–	17	–
	Peru	7	2	–	5	–	6	–
	United States	132	55	44.8 (4.5)	114	88.6 (2.6)	126	97.0 (1.3)
	Overall	238	81	37.3 (3.7)	184	79.7 (2.9)	218	93.7 (1.5)
Externalised disorders								
	Argentina	15	6	–	11	–	13	–
	Brazil	50	23	38.6 (10.1)	38	72.3 (11.1)	43	88.5 (6.2)
	Colombia	28	4	–	19	–	21	–
	Medellin Colombia	10	3	–	7	–	7	–
	Mexico	18	5	–	11	–	14	–
	Peru	20	4	–	12	–	15	–
	United States	253	90	34.9 (3.2)	193	75.0 (3.6)	228	88.5 (2.5)
	Overall	394	135	33.8 (2.8)	291	73.1 (3.0)	341	86.4 (2.1)
Any disorder								
	Argentina	182	72	42.9 (4.4)	130	72.9 (4.8)	158	87.2 (2.8)
	Brazil	332	128	39.8 (3.5)	247	75.2 (2.9)	292	88.9 (2.2)
	Colombia	131	21	19.8 (5.2)	69	58.0 (5.3)	99	79.1 (4.6)
	Medellin Colombia	113	26	22.7 (5.0)	78	64.8 (5.8)	94	81.7 (4.9)
	Mexico	142	29	21.9 (3.6)	82	57.1 (5.0)	110	82.9 (3.2)
	Peru	83	10	12.4 (3.6)	44	51.4 (5.8)	59	71.4 (5.0)
	United States	1008	392	38.6 (1.7)	794	77.4 (1.9)	915	89.4 (1.6)
	Overall	1991	678	35.3 (1.3)	1444	72.8 (1.3)	1727	87.1 (1.0)

WMH, World Mental Health; CIDI, Composite International Diagnostic Interview; s.e., standard error.

– Percentage less than twice the s.e. or sample size < 30.

Analyses performed on the part II sample.

Light treatment was defined as at least four visits in the prior year to any type of provider, or at least two visits and any type of medication, or currently in treatment at the time of the interview. Follow-up treatment was defined as at least two visits in any service sector in the past 12 months or currently in treatment.

Anxiety disorders: panic disorder and/or agoraphobia, specific phobia, social phobia, generalised anxiety disorder, adult separation anxiety disorder and PTSD. Mood disorders: major depressive disorder/dysthymia and bipolar broad. Substance use disorders: alcohol and drug abuse/dependence. Externalised disorders: attention-deficit/hyperactivity disorder and intermittent explosive disorder.

Intermittent explosive disorder was not assessed in Mexico and Medellin and was coded as zero.

Imputed variables for alcohol and drug dependence were used for Colombia, Mexico, Peru and the U.S.

Lifetime ADHD was used in all countries and was coded as zero for those with age > 45 in Colombia, Mexico, Peru and the U.S.

Additional analyses were performed for treatment adequacy, comorbidity and disorder severity. Overall, the greater the severity and comorbidity, the higher the adequacy of treatment (online Supplementary Table 3S-Annex). For example, overall the prevalence of treatment adequacy using a stringent definition for those with one disorder only was 29.5% but increased to 49.3% for those with four+ disorders. By the same token, it was 25.9% among those with a mild disorder to as high as 43.4% for those reporting a severe disorder. Data was too scarce for a detailed description of this trend across study sites.

### Socio-demographic predictors of treatment

We looked at demographic (sex, age, education, marital status and family income) associations for any treatment overall and by the study site (Table 4). Overall, women were more likely than men to receive any treatment; those with less education were less likely to receive any treatment compared to those with the highest education; those previously married were more likely to get any treatment than those married-cohabitating; finally, all those below ‘high family income’ were less likely to receive any treatment.

**Table 4.** Socio-demographic predictors for 12 month service use among WMH respondents with 12 month DSM-IV/CIDI disorders in the WMH-PAHO surveys, country effect *v*. overall effect

	Overall (*n* = 6710)		Argentina (*n* = 568)		Brazil (*n* = 1248)		Colombia (*n* = 847)		Medellin, Colombia (*n* = 530)		Mexico (*n* = 695)		Peru (*n* = 391)		United States (*n* = 2431)	
Variable	aOR^a^	(95% CI)	aOR^a^	(95% CI)	aOR^a^	(95% CI)	aOR^a^	(95% CI)	aOR^a^	(95% CI)	aOR^a^	(95% CI)	aOR^a^	(95% CI)	aOR^a^	(95% CI)
Sex																
Female	1.2*	(1.0–1.5)	1.0	(0.6–1.5)	1.1	(0.8–1.6)	1.0	(0.6–1.7)	0.9	(0.5–1.5)	0.7	(0.5–1.1)	0.9	(0.5–1.8)	1.5*	(1.2–1.8)
Male	1.0	–	1.0	–	1.0	–	1.0	–	1.0	–	1.0	–	1.0	–	1.0	–
*χ*^2^_1_ (*p*-value)	3.9* (0.048)		0.0 (0.937)		0.5 (0.485)		0.0 (0.969)		0.2 (0.680)		1.9 (0.163)		0.0 (0.857)		10.8* (0.001)	
Age																
Age 18–34	0.6	(0.3–1.4)	0.3*	(0.1–1.0)	1.0	(0.3–3.3)	5.2	(0.3–89.1)	2.1	(0.9–4.8)	0.4	(0.1–2.8)	0.4	(0.1–2.9)	2.0	(0.9–4.8)
Age 35–49	1.0	(0.5–2.1)	0.5	(0.2–1.6)	1.0	(0.3–3.0)	4.1	(0.3–64.6)	1.8	(0.9–3.7)	0.6	(0.1–4.1)	0.2	(0.0–1.6)	1.9	(0.9–4.3)
Age 50–64	1.1	(0.5–2.3)	0.7	(0.2–2.4)	1.2	(0.4–3.8)	5.1	(0.4–73.0)	1.0	–	0.5	(0.1–3.6)	0.2	(0.0–2.1)	1.8	(0.8–4.0)
Age ⩾65	1.0	–	1.0	–	1.0	–	1.0	–	1.0	–	1.0	–	1.0	–	1.0	–
*χ*^2^_2–3_ (*p*-value)	20.4* (<0.001)		12.4* (0.006)		1.2 (0.748)		2.1 (0.558)		3.4 (0.184)		4.5 (0.213)		4.5 (0.208)		2.9 (0.414)	
Education																
Low	0.7*	(0.5–1.0)	0.4*	(0.2–1.0)	0.8	(0.4–1.4)	1.2	(0.6–2.5)	1.3	(0.4–4.5)	1.1	(0.5–2.6)	1.5	(0.5–4.6)	1.2	(0.7–1.8)
Low average	0.6*	(0.5–0.8)	0.6	(0.3–1.2)	1.0	(0.5–1.9)	1.7	(0.8–3.5)	1.0	(0.4–2.2)	0.9	(0.5–1.9)	0.7	(0.3–2.0)	1.4	(1.0–2.1)
High average	0.7*	(0.5–0.9)	0.9	(0.5–1.6)	1.2	(0.6–2.2)	0.8	(0.4–1.8)	0.9	(0.5–1.7)	1.0	(0.5–1.8)	0.9	(0.5–1.9)	1.4*	(1.0–1.9)
High	1.0	–	1.0	–	1.0	–	1.0	–	1.0	–	1.0	–	1.0	–	1.0	–
*χ*^2^_3_ (*p*-value)	12.3* (0.007)		6.1 (0.108)		2.4 (0.490)		4.6 (0.201)		0.5 (0.921)		0.3 (0.966)		0.9 (0.824)		5.2 (0.156)	
Marital status																
Married-cohabitating	1.0	–	1.0	–	1.0	–	1.0	–	1.0	–	1.0	–	1.0	–	1.0	–
Previously married	1.3*	(1.0–1.7)	0.6	(0.3–1.1)	0.7	(0.4–1.0)	1.3	(0.7–2.3)	1.9	(0.9–3.8)	1.0	(0.5–1.9)	0.9	(0.5–1.7)	1.1	(0.8–1.5)
Never married	1.2	(1.0–1.5)	1.3	(0.8–2.0)	0.8	(0.5–1.4)	0.8	(0.5–1.5)	0.7	(0.4–1.3)	1.8	(1.0–3.4)	1.0	(0.6–1.7)	0.9	(0.7–1.2)
*χ*^2^_2_ (*p*-value)	6.8* (0.033)		5.7 (0.058)		3.6 (0.166)		1.5 (0.472)		6.6* (0.038)		3.9 (0.144)		0.2 (0.900)		1.2 (0.563)	
Income																
Low	0.7*	(0.5–0.8)	0.6	(0.3–1.3)	1.5	(0.9–2.5)	0.6	(0.3–1.3)	1.0	(0.5–1.9)	1.8	(0.9–3.5)	0.7	(0.4–1.2)	1.3	(1.0–1.9)
Low average	0.6*	(0.5–0.8)	0.9	(0.4–2.0)	1.7*	(1.1–2.8)	0.4*	(0.2–0.8)	0.8	(0.4–1.7)	1.8	(1.0–3.1)	0.8	(0.4–1.6)	1.5*	(1.0–2.1)
High average	0.7*	(0.5–0.9)	1.1	(0.5–2.5)	1.5	(0.9–2.3)	0.9	(0.5–1.6)	0.5	(0.2–1.1)	1.9	(1.0–3.5)	0.6	(0.2–1.5)	1.3	(0.9–1.9)
High	1.0	–	1.0	–	1.0	–	1.0	–	1.0	–	1.0	–	1.0	–	1.0	–
*χ*^2^_3_ (*p*-value)	18.1* (<0.001)		3.9 (0.272)		5.1 (0.163)		9.6* (0.022)		4.0 (0.266)		5.2 (0.156)		2.5 (0.474)		5.1 (0.168)	

*Note*: each row shows a separate logistic regression model with 12 month service use as the outcome variable, controlling for the other predictor variables (rows), survey and all predictor-by-survey interaction dummies. The second column shows the overall adjusted predictor variable effect. The survey columns show to what extent the survey-specific adjusted predictor variable effect deviates from the overall adjusted predictor variable effect. For example, the survey-specific effect for females (*v*. males) in the U.S. can be obtained by multiplying the aOR  = 1.2 (the overall effect) by the aOR  = 1.5 (the country-specific deviation), i.e., aOR  = 1.8.

Reference categories are denoted as 1.0. Age groups 50–64 and 65+ were collapsed for Medellin, Colombia.

The degrees of freedom for each chi-square test are based on the number of groups available in each main category.

Models include controls for groups of 12 month DSM-IV/WMH CIDI disorders (any anxiety, any mood, any substance and any externalised).

Intermittent explosive disorder was not assessed in Mexico and Medellin and was coded as zero. Imputed variables for alcohol and drug dependence were used for Colombia, Mexico, Peru and the U.S. Lifetime ADHD was used in all countries and was coded as zero for those with age > 45 in Colombia, Mexico, Peru and the U.S.

a Data are given as adjusted odd ratios (95% confidence interval) unless otherwise indicated.

*Significant at *p*  =  0.05, two-sided test.

Some differences were apparent when looking at statistically significant deviations from the overall estimate by the study site. Firstly, women in the U.S. were 1.5 times more likely than the overall estimate to use any service. Compared to the overall estimate, 18–34 year-olds in Argentina were even less likely to use any service. With the exception of those with lower education in Argentina, which showed lower likelihood of service use, and of those with high education in the U.S having greater service use, no other associations were found in the other sites for educational attainment and the likelihood of any service use. A low-average family income was associated with higher service use in the U.S. and Brazil and lower service use in Colombia-national.

### Socio-demographic predictors of 12 month treatment adequacy

Table 5 presents associations of demographic variables with the light definition of 12 month treatment adequacy, overall and by the study site. Overall, those with less education and those with lower income were less likely to obtain adequate treatment compared to those with the highest levels of education and income.

**Table 5.** Socio-demographic predictors for adequacy of treatment (light definition) among WMH respondents with 12 month DSM-IV/CIDI disorders in the WMH-PAHO surveys, country effect *v*. overall effect

Variable	Overall (*n* = 6710)		Argentina (*n* = 568)		Brazil (*n* = 1248)		Colombia (*n* = 847)		Medellin, Colombia (*n* = 530)		Mexico (*n* = 695)		Peru (*n* = 391)		United States (*n* = 2431)	
	aOR^a^	(95% CI)	aOR^a^	(95% CI)	aOR^a^	(95% CI)	aOR^a^	(95% CI)	aOR^a^	(95% CI)	aOR^a^	(95% CI)	aOR^a^	(95% CI)	aOR^a^	(95% CI)
Sex																
Female	1.1	(0.9–1.5)	0.9	(0.5–1.7)	1.1	(0.7–1.7)	0.9	(0.4–1.7)	0.8	(0.4–1.5)	0.9	(0.5–1.7)	1.0	(0.4–2.5)	1.5*	(1.1–2.0)
Male	1.0	–	1.0	–	1.0	–	1.0	–	1.0	–	1.0	–	1.0	–	1.0	–
*χ*^2^_1_ (*p*-value)	1.0 (0.320)		0.0 (0.842)		0.2 (0.655)		0.2 (0.635)		0.4 (0.535)		0.0 (0.856)		0.0 (0.975)		7.0* (0.008)	
Age																
Age 18–34	0.6	(0.3–1.3)	0.2*	(0.0–0.7)	2.2	(0.7–6.9)	0.9	(0.4–2.5)	1.9	(0.7–5.3)	0.6	(0.1–4.1)	1.5	(0.1–24.1)	1.7	(0.7–4.1)
Age 35–49	0.9	(0.4–1.9)	0.4	(0.1–1.6)	2.2	(0.8–6.4)	0.6	(0.2–1.5)	1.1	(0.5–2.3)	1.0	(0.1–7.7)	0.9	(0.1–13.0)	1.8	(0.8–4.3)
Age 50–64	1.2	(0.5–2.7)	0.5	(0.1–1.7)	2.3	(0.8–6.4)	1.0	–	1.0	–	0.9	(0.1–5.8)	0.8	(0.0–13.7)	1.4	(0.6–3.2)
Age ⩾65	1.0	–	1.0	–	1.0	–	1.0	–	1.0	–	1.0	–	1.0	–	1.0	–
*χ*^2^_2–3_ (*p*-value)	17.9* (<0.001)		12.5* (0.006)		2.4 (0.496)		1.6 (0.441)		2.2 (0.336)		5.2 (0.155)		2.0 (0.575)		3.0 (0.389)	
Education																
Low	0.6*	(0.4–0.9)	0.3*	(0.1–0.7)	1.0	(0.5–2.0)	0.8	(0.3–1.9)	1.1	(0.2–5.6)	0.8	(0.3–1.9)	2.7	(0.7–10.5)	1.8*	(1.1–3.0)
Low average	0.6*	(0.4–0.8)	0.5	(0.3–1.1)	1.0	(0.5–2.2)	1.3	(0.6–2.9)	1.2	(0.5–3.1)	0.6	(0.3–1.5)	–	–	1.8*	(1.2–2.8)
High average	0.7*	(0.5–0.9)	0.5*	(0.3–1.0)	1.3	(0.7–2.5)	0.8	(0.3–2.0)	0.8	(0.4–1.6)	0.9	(0.4–1.8)	1.6	(0.9–2.8)	1.5*	(1.1–2.2)
High	1.0	–	1.0	–	1.0	–	1.0	–	1.0	–	1.0	–	1.0	–	1.0	–
*χ*^2^_2–3_ (*p*-value)	11.5* (0.009)		16.6* (<0.001)		1.3 (0.717)		1.3 (0.718)		1.7 (0.630)		1.1 (0.766)		3.6 (0.168)		9.6* (0.023)	
Marital status																
Married-cohabitating	1.0	–	1.0	–	1.0	–	1.0	–	1.0	–	1.0	–	1.0	–	1.0	–
Previously married	1.3	(1.0–1.7)	0.6	(0.4–1.2)	0.7	(0.4–1.0)	1.0	(0.4–2.5)	2.3*	(1.1–4.9)	0.9	(0.5–1.8)	1.0	(0.4–2.2)	1.1	(0.8–1.6)
Never married	1.2	(0.9–1.6)	1.6	(0.8–3.1)	0.7	(0.5–1.2)	0.8	(0.4–1.8)	0.6	(0.3–1.3)	2.2*	(1.1–4.4)	0.8	(0.4–1.6)	1.0	(0.7–1.5)
*χ*^2^_2_ (*p*-value)	4.9 (0.085)		5.2 (0.073)		4.4 (0.108)		0.3 (0.881)		10.5* (0.005)		6.4* (0.041)		0.5 (0.760)		0.3 (0.882)	
Income																
Low	0.7*	(0.5–0.9)	0.7	(0.3–1.6)	1.6	(0.9–2.7)	0.7	(0.3–1.6)	1.1	(0.6–2.3)	1.8	(0.8–4.3)	0.5*	(0.3–0.9)	1.2	(0.8–1.7)
Low average	0.6*	(0.5–0.9)	1.2	(0.5–2.9)	1.6	(0.9–2.6)	0.4*	(0.2–0.8)	1.0	(0.4–2.4)	1.7	(0.8–3.6)	0.7	(0.3–1.7)	1.2	(0.8–1.8)
High average	0.7*	(0.5–0.9)	1.5	(0.6–3.6)	1.6	(0.9–2.9)	0.5	(0.2–1.1)	0.9	(0.4–2.0)	1.9	(0.8–4.7)	0.4	(0.2–1.1)	1.2	(0.8–1.8)
High	1.0	–	1.0	–	1.0	–	1.0	–	1.0	–	1.0	–	1.0	–	1.0	–
*χ*^2^_3_ (*p*-value)	11.7* (0.009)		4.3 (0.234)		4.1 (0.256)		7.8 (0.051)		0.4 (0.931)		3.0 (0.396)		7.4 (0.060)		1.5 (0.682)	

*Note*: each row shows a separate logistic regression model with 12 month service use as the outcome variable, controlling for the other predictor variables (rows), survey and all predictor-by-survey interaction dummies. The second column shows the overall adjusted predictor variable effect; the survey columns show to what extent the survey-specific adjusted predictor variable effect deviates from the overall adjusted predictor variable effect. For example, the survey-specific effect for females (*v*. males) in the U.S. can be obtained by multiplying the aOR  =  1.1 (the overall effect) by the aOR  = 1.5 (the country-specific deviation), i.e., aOR  =  1.65.

Reference categories are denoted as 1.0. Age groups 50–64 and 65+ were collapsed for Colombia and Medellin, Colombia. The low average category for Peru was excluded due to cells with zero-count.

The degrees of freedom for each chi-square test are based on the number of groups available in each main category.

Models include controls for groups of 12 month DSM-IV/WMH CIDI disorders (any anxiety, any mood, any substance and any externalised).

Intermittent explosive disorder was not assessed in Mexico and Medellin and was coded as zero. Imputed variables for alcohol and drug dependence were used for Colombia, Mexico, Peru and the U.S. Lifetime ADHD was used in all countries, and was coded as zero for those with age > 45 in Colombia, Mexico, Peru and the U.S.

a Data are given as adjusted odd ratios (95% confidence interval) unless otherwise indicated.

*Significant at *p*  =  0.05, two-sided test.

Few significant deviations from the overall estimates by the study site were observed: females in the U.S. were more likely to receive adequate treatment and the youngest group in Argentina was even less likely to receive adequate treatment. Lower education was associated with lower treatment adequacy in Argentina, while those with lower education in the U.S. had higher probability of adequate treatment compared to the overall estimate. The marital status was only associated with treatment adequacy in Medellin-Colombia and Mexico, where those previously married and those never married, respectively, were more likely to receive adequate treatment. Overall, lower family income was associated with treatment adequacy; in Colombia-national and Peru, those in low-average and those in low-income groups, respectively, were less likely to receive adequate treatment.

Finally, we looked at predictors of adequate treatment among those with any disorder and any service use. No significant association was found overall. Among those in treatment, higher significant deviations from the overall estimate were observed among the two youngest groups in Brazil; similarly, the two lower education groups in the U.S. had higher probability of adequate treatment, while in Argentina the low and high-average education groups were even less likely to receive adequate treatment (online Supplementary Table 4S-Annex).

## Discussion

### Limitations

First, the WHO WMH surveys exclude people who are homeless or institutionalised and, in most surveys, do not represent people living in rural areas. Some clinically important disorders such as schizophrenia were not assessed in WMH surveys, but most respondents would still meet criteria for comorbid anxiety, mood, or substance disorders, and are therefore captured in our analyses. Another related limitation is that the exact disorders assessed also varied across surveys because some were felt a priori to have low relevance in some countries. This set of limitations is likely to have caused us to underestimate the magnitude of unmet needs for any mental health treatment and minimally adequate treatment. Some of the countries included here are among the richest in the region and the overall prevalences reported here are likely to be above the ones for some of the poorest countries in the Americas. Without corroborating data on service use, we cannot study the validity of self-reported treatment use in the WMHS. Potentially biased recall of mental health service use is thereby a limitation.

Our definitions of minimally adequate treatment, which focus predominantly on treatment duration may differ from others in use and, to our knowledge, their relationships with important clinical outcomes have not been studied. Nevertheless, our sensitivity analyses using more light or stringent definitions can help to formulate best and worst-case scenarios for the participating sites. While we included an ample range of mental disorders, participant characteristics and service types, we did not include other potentially important variables, such as attitudinal barriers, the characteristics of providers, insurance coverage or costs. Finally, we cannot conclude that factors associated with receiving any treatment or minimally adequate treatment are causally related because of the study's cross-sectional nature.

### Findings

Our results regarding the large-treatment gap in these survey sites from the Americas is in line with what have been reported for other parts of the world (Wang *et al*., 2007). Our result of a mean of 27.6% (that ranged from 39.7% in the USA to 13.1% in Colombia-national) is a little lower than the mean of 29.0% among a larger group of 25 countries recently reported, that included six of the seven sites considered here (Evans-Lacko *et al*., 2018). Nevertheless, with the exception of the larger prevalence of service use in the USA, all other six sites in our region had rates that were below the 29.0% mean, suggesting that most countries in the region have to struggle even further with the challenge of limited resources for mental health treatment. Interestingly, with the exception of Peru, rates were higher in the two high-income sites, followed by the three upper-middle income sites and the lower-middle income sites in Colombia-national. The high rate of service use in Peru, nevertheless, has to be taken with caution because the survey included only large urban areas of that country that, most likely, concentrate mental health resources. Treatment adequacy, with some minor exceptions, shows the same tendency with high-income sites performing better, followed by upper-middle and lower-middle income sites. This trend by economic ranking was also noted for a larger number of countries in the WMH Surveys that focused on any mental disorder (Evans-Lacko *et al*., 2018), and also in analyses for specific disorders, such as substance use disorders (Degenhardt *et al*., 2017) and major depressive disorder (Thornicroft *et al*., 2017). Taken at face value, it could be concluded that given the availability of more financial resources, the mental health gap in the region would decrease. Nevertheless, at least two other issues should be considered. First, the distribution of resources for mental health among service providers in the region varied importantly. If we consider the prevalence of services provided by general medical practitioners (mean of 12.9% for any disorder as per Table 1) as an example of a gate-keeper strategy, this prevalence was 21.8% for the USA, almost half that in Argentina (11.1%), and even less in Brazil (8.3%), Mexico (5.9%), Colombia-Medellin (6.9%), Colombia-national (5.5%) and Peru (4.8%). The allocation and strategy for patients entering the mental health treatment system in the region clearly has room for improvement. Secondly, if we use the stringent definition to measure the best quality of care among the most trained professional (i.e., the psychiatrist) this ranged from about one in every three patients with any disorder being adequately treated in Brazil to a low of one in every 20 patients in Peru (online Supplementary Table 2S-Annex). Again, while in all sites there is ample room for improving mental health care, a regional effort to upgrade and to make more uniform the quality of care is needed.

With regard to socio-demographic factors for all sites combined, being female was associated with higher likelihood of treatment. A prior study of treatment use and treatment adequacy in 17 WMH countries found similar results (Wang *et al*., 2007). Sex was associated with any 12 month service use in 10 of the 17 countries, with females being more likely to receive services in all 10. Similar results in other studies have also found less help seeking for mental health concerns in males than females (Addis and Mahalik, 2003; Judd *et al*., 2008). These overall results, nevertheless, did not hold across every study-site. Females, in certain cultures, may be more willing to share and identify psychological distress whereas traditional masculine roles have been associated with more negative attitudes towards emotional disclosure and help-seeking (Seidler *et al*., 2016). In this study, age and marital status were relevant factors in some, but not all survey sites. In Argentina, the youngest age group had lower likelihood of receiving services while in the rest of the sites there was no increase or decrease in service use compared to the overall estimates. Similar to that found in five of 17 WMH countries those married/cohabitating were less likely to receive services than the unmarried perhaps suggesting that relationship discord, lack of social supports or loneliness may facilitate or trigger help-seeking (Wang *et al*., 2007).

A great interest exists in whether socio-economic disadvantages are leading factors in the treatment gap for mental disorders (Evans-Lacko *et al*., 2018). Here, using two measures of social disadvantage (educational attainment and family income), our results seem to confirm this. Overall, the lower the educational attainment the less likely a respondent was to have received any treatment. Taken together, these results suggest that the mental health treatment gap in the region may be a part of social inequities that abound in countries like Argentina (42.7 World Bank Gini index in 2014), Brazil (51.3 in 2015), Colombia (51.1 in 2015), Mexico (48.2 in 2014), Peru (44.3 in 2015) and even the USA (41.0 in 2013) (The World Bank, 2018). Reversing this trend is a daunting task that likely goes well beyond the funding of health care. Nevertheless, as mentioned above, a better organization and allocation of the scarce resources seems doable. Examples of local and national initiatives to make mental health care more horizontal (less pyramidal) and close to the population in need have been undertaken in some countries of the region (Mateus *et al*., 2008; Costa *et al*., 2011).

## Conclusion

We found large unmet needs among those with mental disorders, extensive underutilization of mental health services and provision of services that sometimes lack adequacy. People with social disadvantages tend to be more affected by these treatment gaps. Creating and promoting services that are more accessible, especially for those with fewer resources, are urgently needed.

## Supplementary material

For supplementary material accompanying this paper visit https://doi.org/10.1017/S2045796019000477. click here to view supplementary material

## Data

Data will not be shared due to private funding for some of the countries.
