# Supplementary material for: Twelve-month mental health service use in six countries of the Americas: A regional report from the World Mental Health Surveys
Source: Epidemiol Psychiatr Sci. 2019 Aug 27;29:e53. doi: 10.1017/S2045796019000477 (PMC8061239; doi:10.1017/S2045796019000477)
Supplement: Supplementary file 1 [file S2045796019000477sup001.docx]

| **Annex - Table 1S:** WMH sample characteristics by World Bank income categories*^a^* | | | | | | | |
| --- | --- | --- | --- | --- | --- | --- | --- |
|  |  |  |  |  | **Sample size** | |  |
| **Country by income category** | **Survey***^b^* | **Sample characteristics*^c^*** | **Field dates** | **Age range** | **Part I** | **Part II** | **Response rate*^d^*** |
| **I. Low and lower middle income countries** | | |  |  |  |  |  |
|  |  |  |  |  |  |  |  |
| Colombia | NSMH | All urban areas of the country -approximately 73% of the total national population | 2003 | 18-65 | 4,426 | 2,381 | 87.7 |
| Peru | EMSMP | Five urban areas of the country – approximately 38% of the total national population. | 2004-5 | 18-65 | 3,930 | 1,801 | 90.2 |
| TOTAL |  |  |  |  | (8,356) | (4,182) | 88.9 |
| **II. Upper-middle income countries** | | |  |  |  |  |  |
|  |  |  |  |  |  |  |  |
| Brazil - São Paulo | São Paulo Megacity | São Paulo metropolitan area. | 2005-8 | 18-93 | 5,037 | 2,942 | 81.3 |
| Colombia - Medellín | MMHHS | Medellín metropolitan area | 2011-12 | 19-65 | 3,261 | 1,673 | 97.2 |
|  |  |  |  |  |  |  |  |
| Mexico | M-NCS | All urban areas of the country – approximately 75% of the total national population. | 2001-2 | 18-65 | 5,782 | 2,362 | 76.6 |
| **TOTAL** |  |  |  |  | (14,080) | (6,977) | 82.3 |
| **III. High-income countries** | | |  |  |  |  |  |
|  |  |  |  |  |  |  |  |
| Argentina | AMHES | Eight largest urban areas of the country – approximately 50% of the total national population | 2015 | 18-98 | 3,927 | 2,116 | 77.3 |
|  |  |  |  |  |  |  |  |
| United States | NCS-R | Nationally representative | 2001-3 | 18-99 | 9,282 | 5,692 | 70.9 |
| **TOTAL** |  |  |  |  | (13,209) | (7,808) | 72.7 |
| **IV. TOTAL** |  |  |  |  | (35,645) | (18,967) | 79.8 |

*^a^* The World Bank (2012) Data. Accessed May 12, 2012 at: <http://data.worldbank.org/country>. Some of the WMH countries have moved into new income categories since the surveys were conducted. The income groupings above reflect the status of each country at the time of data collection. The current income category of each country is available at the preceding URL.

*^b^* NSMH (The Colombian National Study of Mental Health); EMSMP (La Encuesta Mundial de Salud Mental en el Peru); MMHHS (Medellín Mental Health Household Study); M-NCS (The Mexico National Comorbidity Survey); AMHES (Argentina Mental Health Epidemiologic Survey); NCS-R (The US National Comorbidity Survey Replication).

*^c^* Most WMH surveys are based on stratified multistage clustered area probability household samples in which samples of areas equivalent to counties or municipalities in the US were selected in the first stage followed by one or more subsequent stages of geographic sampling (e.g., towns within counties, blocks within towns, households within blocks) to arrive at a sample of households, in each of which a listing of household members was created and one or two people were selected from this listing to be interviewed. No substitution was allowed when the originally sampled household resident could not be interviewed.

*^d^* The response rate is calculated as the ratio of the number of households in which an interview was completed to the number of households originally sampled, excluding from the denominator households known not to be eligible either because of being vacant at the time of initial contact or because the residents were unable to speak the designated languages of the survey. The weighted average response rate is 79.8%.

| **Annex - Table 2S: Adequacy of treatment ('stringent' definition), by service sectors, among WMH respondents with any 12-month DSM-IV/CIDI disorders who received any 12 month treatment in the PAHO Region (n=1991).** | | | | | | | | | | |
| --- | --- | --- | --- | --- | --- | --- | --- | --- | --- | --- |
|  | | **No. of respondents** | **Psychiatrist** | **Other Mental Health Specialist** | **Any Mental Health Specialist** | **General Medical** | **Any Health Care** | **Human Services** | **CAM** | **Any Service Use** |
|  | **Survey** | **Unweighted n** | **% (SE)** | **% (SE)** | **% (SE)** | **% (SE)** | **% (SE)** | **% (SE)** | **% (SE)** | **% (SE)** |
| Any disorder |  |  |  |  |  |  |  |  |  |  |
|  | Argentina | 182 | 16.8 (3.3) | 34.5 (5.3) | 39.9 (5.0) | 12.4 (3.9) | 42.9 (4.4) | -- | 2.7 (1.4) | 42.9 (4.4) |
|  | Brazil | 332 | 29.6 (3.9) | 20.6 (2.4) | 37.6 (3.5) | 8.6 (1.2) | 39.8 (3.5) | 3.8 (1.1) | 5.3 (1.0) | 39.8 (3.5) |
|  | Colombia | 131 | 7.7 (2.7) | 8.1 (4.0) | 14.2 (4.5) | -- | 19.8 (5.2) | 0 (0) | 0 (0) | 19.8 (5.2) |
|  | Medellin Colombia | 113 | 17.0 (4.1) | 15.1 (4.5) | 22.1 (5.0) | -- | 22.7 (5.0) | -- | -- | 22.7 (5.0) |
|  | Mexico | 142 | 7.4 (2.8) | 11.5 (2.8) | 17.0 (3.1) | 7.7 (2.5) | 21.9 (3.6) | 0 (0) | -- | 21.9 (3.6) |
|  | Peru | 83 | 5.3 (2.3) | -- | 11.5 (3.8) | -- | 12.4 (3.6) | -- | -- | 12.4 (3.6) |
|  | United States | 1008 | 19.9 (1.6) | 28.1 (1.7) | 34.2 (1.6) | 22.2 (1.4) | 38.6 (1.7) | 7.2 (0.9) | 7.4 (0.9) | 38.6 (1.7) |
|  | Overall | 1991 | 19.2 (1.2) | 23.7 (1.2) | 31.6 (1.2) | 15.6 (1.0) | 35.3 (1.3) | 5.0 (0.6) | 5.6 (0.6) | 35.3 (1.3) |
| *Abbreviations: CAM, complementary and alternative medicine; WMH, World Mental Health; CIDI, Composite International Diagnostic Interview; SE, Standard Error* | | | | | | | | | | |
| *Analyses performed on part II sample* | | | | | | | | | | |
| *-- Percentage less than twice the SE or sample size < 30* | | | | | | | | | | |
| *Interminent explosive disorder was not assessed in Mexico and Medellin, were coded as zero* | | | | | | | | | | |
| *Imputed variables for alcohol and drug dependence were used for Colombia, Mexico, Peru and the U.S.* | | | | | | | | | | |
| *Lifetime ADHD was used in all countries, and was coded as zero for those with age > 45 in Colombia, Mexico, Peru and the U.S.* | | | | | | | | | | |

| **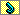****Annex - Table 3S: Adequacy of treatment among WMH respondents with any 12-month DSM-IV/CIDI disorders with any service use, by number of disorders and severity, by survey in the PAHO region (n=1991).** | | | | | | | | | |
| --- | --- | --- | --- | --- | --- | --- | --- | --- | --- |
|  |  | **Number of disorders / Severity** | **No. of respondents with disorder** | **Minimally adequate treatment ('stringent' definition)** | | **Minimally adequate treatment ('light' definition)** | | **Follow-up treatment ('very light' definition)** | |
| **Variable** | **Survey** |  | **Unweighted n** | **Unweighted n** | **% (SE)** | **Unweighted n** | **% (SE)** | **Unweighted n** | **% (SE)** |
| Number of disorders |  |  |  |  |  |  |  |  |  |
|  | Argentina | 1 | 100 | 41 | 40.5 (5.8) | 71 | 71.3 (7.3) | 88 | 88.6 (3.5) |
|  | Argentina | 2 | 42 | 17 | 54.7 (9.8) | 31 | 80.7 (5.5) | 36 | 87.9 (4.5) |
|  | Argentina | 3 | 18 | 4 | -- | 11 | -- | 14 | -- |
|  | Argentina | 4+ | 22 | 10 | -- | 17 | -- | 20 | -- |
|  | Brazil | 1 | 148 | 45 | 30.9 (4.3) | 104 | 70.3 (4.1) | 129 | 86.0 (3.7) |
|  | Brazil | 2 | 74 | 32 | 44.7 (8.0) | 54 | 74.7 (6.6) | 66 | 91.7 (3.7) |
|  | Brazil | 3 | 57 | 23 | 40.5 (8.3) | 46 | 81.5 (5.9) | 52 | 94.8 (2.6) |
|  | Brazil | 4+ | 53 | 28 | 57.6 (8.3) | 43 | 83.9 (7.2) | 45 | 86.0 (7.0) |
|  | Colombia | 1 | 62 | 12 | 20.6 (8.0) | 27 | 49.5 (8.8) | 41 | 67.6 (8.1) |
|  | Colombia | 2 | 41 | 5 | 16.5 (7.7) | 26 | 70.9 (7.4) | 35 | 91.6 (4.4) |
|  | Colombia | 3 | 16 | 2 | -- | 8 | -- | 12 | -- |
|  | Colombia | 4+ | 12 | 2 | -- | 8 | -- | 11 | -- |
|  | Medellin Colombia | 1 | 56 | 11 | 18.3 (6.0) | 35 | 62.4 (8.2) | 44 | 80.4 (7.7) |
|  | Medellin Colombia | 2 | 28 | 7 | -- | 23 | -- | 26 | -- |
|  | Medellin Colombia | 3 | 16 | 4 | -- | 11 | -- | 13 | -- |
|  | Medellin Colombia | 4+ | 13 | 4 | -- | 9 | -- | 11 | -- |
|  | Mexico | 1 | 76 | 15 | 21.2 (5.2) | 43 | 54.4 (7.5) | 57 | 78.5 (5.2) |
|  | Mexico | 2 | 38 | 7 | 25.8 (9.4) | 22 | 67.2 (9.4) | 29 | 88.0 (4.7) |
|  | Mexico | 3 | 18 | 5 | -- | 11 | -- | 16 | -- |
|  | Mexico | 4+ | 10 | 2 | -- | 6 | -- | 8 | -- |
|  | Peru | 1 | 46 | 3 | -- | 22 | 44.7 (8.0) | 29 | 63.3 (5.9) |
|  | Peru | 2 | 21 | 7 | -- | 15 | -- | 18 | -- |
|  | Peru | 3 | 12 | 0 | -- | 5 | -- | 9 | -- |
|  | Peru | 4+ | 4 | 0 | -- | 2 | -- | 3 | -- |
|  | United States | 1 | 388 | 135 | 33.6 (2.8) | 293 | 74.7 (2.5) | 341 | 87.1 (2.2) |
|  | United States | 2 | 264 | 90 | 34.3 (2.6) | 207 | 76.1 (4.0) | 242 | 89.3 (3.1) |
|  | United States | 3 | 153 | 65 | 41.5 (6.1) | 124 | 77.1 (5.6) | 140 | 88.5 (4.1) |
|  | United States | 4+ | 203 | 102 | 52.0 (3.5) | 170 | 84.7 (2.7) | 192 | 94.8 (1.5) |
|  | Overall | 1 | 876 | 262 | 29.5 (1.8) | 595 | 68.3 (1.9) | 729 | 83.5 (1.5) |
|  | Overall | 2 | 508 | 165 | 36.2 (2.4) | 378 | 75.5 (2.7) | 452 | 89.7 (2.0) |
|  | Overall | 3 | 290 | 103 | 36.1 (4.2) | 216 | 72.2 (3.8) | 256 | 87.4 (2.7) |
|  | Overall | 4+ | 317 | 148 | 49.3 (3.0) | 255 | 81.6 (2.5) | 290 | 92.5 (1.6) |
| Severity |  |  |  |  |  |  |  |  |  |
|  | Argentina | Severe | 61 | 25 | 37.2 (9.0) | 46 | 74.3 (6.7) | 52 | 82.2 (5.5) |
|  | Argentina | Moderate | 72 | 31 | 49.5 (6.3) | 53 | 74.8 (6.7) | 65 | 92.1 (3.4) |
|  | Argentina | Mild | 49 | 16 | 38.5 (9.2) | 31 | 68.6 (9.2) | 41 | 84.5 (6.1) |
|  | Brazil | Severe | 170 | 75 | 45.6 (5.8) | 136 | 82.1 (2.9) | 155 | 92.0 (2.4) |
|  | Brazil | Moderate | 97 | 30 | 36.8 (5.7) | 70 | 77.4 (4.8) | 83 | 90.4 (3.4) |
|  | Brazil | Mild | 65 | 23 | 29.1 (5.1) | 41 | 52.9 (7.1) | 54 | 78.2 (7.0) |
|  | Colombia | Severe | 54 | 11 | 23.1 (8.5) | 38 | 75.6 (6.0) | 49 | 92.6 (3.5) |
|  | Colombia | Moderate | 47 | 7 | 21.7 (10.5) | 21 | 50.7 (10.1) | 31 | 73.1 (7.9) |
|  | Colombia | Mild | 30 | 3 | -- | 10 | 23.4 (8.7) | 19 | 53.2 (10.8) |
|  | Medellin Colombia | Severe | 48 | 17 | 37.0 (8.5) | 40 | 77.3 (7.4) | 44 | 86.0 (6.5) |
|  | Medellin Colombia | Moderate | 37 | 6 | 13.2 (5.6) | 25 | 60.3 (10.8) | 31 | 80.8 (10.4) |
|  | Medellin Colombia | Mild | 28 | 3 | -- | 13 | -- | 19 | -- |
|  | Mexico | Severe | 52 | 8 | 11.3 (4.5) | 28 | 49.9 (8.4) | 40 | 85.5 (4.2) |
|  | Mexico | Moderate | 53 | 13 | 28.6 (6.3) | 33 | 62.2 (7.5) | 41 | 76.6 (6.7) |
|  | Mexico | Mild | 37 | 8 | 27.1 (8.8) | 21 | 60.1 (9.4) | 29 | 86.5 (6.0) |
|  | Peru | Severe | 28 | 5 | -- | 18 | -- | 24 | -- |
|  | Peru | Moderate | 32 | 2 | -- | 16 | 54.0 (9.5) | 20 | 64.7 (10.2) |
|  | Peru | Mild | 23 | 3 | -- | 10 | -- | 15 | -- |
|  | United States | Severe | 385 | 192 | 50.8 (2.8) | 329 | 85.1 (2.2) | 362 | 93.2 (1.7) |
|  | United States | Moderate | 395 | 140 | 34.5 (2.5) | 298 | 73.6 (2.4) | 357 | 88.8 (2.0) |
|  | United States | Mild | 228 | 60 | 26.3 (2.9) | 167 | 71.7 (3.5) | 196 | 84.6 (2.8) |
|  | Overall | Severe | 798 | 333 | 43.4 (2.2) | 635 | 80.5 (1.6) | 726 | 91.5 (1.2) |
|  | Overall | Moderate | 733 | 229 | 33.0 (2.0) | 516 | 71.1 (1.9) | 628 | 86.5 (1.5) |
|  | Overall | Mild | 460 | 116 | 25.9 (2.2) | 293 | 63.1 (2.6) | 373 | 81.0 (2.1) |
| *Abbreviations: WMH, World Mental Health; CIDI, Composite International Diagnostic Interview; SE, Standard Error* | | | | | | | | | |
| *-- Percentage less than twice the SE or sample size < 30* | | | | | | | | | |
| *Analyses performed on part II sample* | | | | | | | | | |
| *Light treatment was defined as at least 4 visits in the prior year to any type of provider, or at least 2 visits and any type of medication, or currently in treatment at the time of the interview. Follow-up treatment was defined as at least 2 visits in any service sector in the past 12 months or currently in treatment* | | | | | | | | | |
| *Anxiety disorders: Panic disorder and/or agoraphobia, Specific phobia, Social phobia, Generalized anxiety disorder, Adult separation anxiety disorder and PTSD; Mood disorders: Major depressive disorder/Dysthymia and Bipolar broad; Substance use disorders: Alcohol and drug abuse/dependence; Externalized disorders: Attention-deficit/hyperactivity disorder and Intermittent explosive disorder.* | | | | | | | | | |
| *Intermittent explosive disorder was not assessed in Mexico and Medellin, were coded as zero* | | | | | | | | | |
| *Imputed variables for alcohol and drug dependence were used for Colombia, Mexico, Peru and the U.S.* | | | | | | | | | |
| *Lifetime ADHD was used in all countries and was coded as zero for those with age > 45 in Colombia, Mexico, Peru and the U.S.* | | | | | | | | | |

| **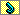Annex - Table 4S: Socio-demographic predictors for adequacy of treatment (light definition) among WMH respondents with 12-month DSM-IV/CIDI disorders and any service use in the WMH-PAHO Surveys, country effect vs. overall effect.** | | | | | | | | | | | | | | | | | |
| --- | --- | --- | --- | --- | --- | --- | --- | --- | --- | --- | --- | --- | --- | --- | --- | --- | --- |
| **Variable** | | **Overall (n = 1991)** | | **Argentina (n = 182)** | | **Brazil (n = 332)** | | **Colombia (n = 131)** | | **Medellin, Colombia (n = 113)** | | **Mexico (n = 142)** | | **Peru (n = 83)** | | **United States (n = 1008)** | |
|  | | **aOR^a^** | **(95% CI)** | **aOR^a^** | **(95% CI)** | **aOR^a^** | **(95% CI)** | **aOR^a^** | **(95% CI)** | **aOR^a^** | **(95% CI)** | **aOR^a^** | **(95% CI)** | **aOR^a^** | **(95% CI)** | **aOR^a^** | **(95% CI)** |
| Sex |  |  |  |  |  |  |  |  |  |  |  |  |  |  |  |  |  |
|  | Female | 1.0 | (0.7-1.4) | 0.7 | (0.4-1.5) | 0.8 | (0.4-1.7) | 0.8 | (0.3-2.2) | 1.0 | (0.3-3.0) | 1.9 | (0.8-4.6) | 0.9 | (0.3-2.7) | 1.2 | (0.7-1.8) |
|  | Male | 1.0 | - | 1.0 | - | 1.0 | - | 1.0 | - | 1.0 | - | 1.0 | - | 1.0 | - | 1.0 | - |
|  | χ^2^_1_ (p-value) | 0.0 (0.870) | | 0.7 (0.389) | | 0.2 (0.620) | | 0.2 (0.665) | | 0.0 (0.982) | | 1.9 (0.170) | | 0.0 (0.883) | | 0.5 (0.493) | |
| Age |  |  |  |  |  |  |  |  |  |  |  |  |  |  |  |  |  |
|  | Age 18-34 | 0.7 | (0.3-1.9) | 0.2 | (0.0-1.3) | 5.2* | (1.1-25.1) | 1.2 | (0.4-3.4) | 0.6 | (0.1-3.1) | 0.8 | (0.1-6.9) | 3.1 | (0.2-53.5) | 0.7 | (0.2-2.2) |
|  | Age 35-49 | 0.8 | (0.3-2.0) | 0.6 | (0.1-2.7) | 4.8* | (1.2-19.1) | 0.9 | (0.3-2.5) | 0.2* | (0.0-0.9) | 1.0 | (0.1-9.3) | 2.5 | (0.2-31.9) | 0.9 | (0.3-2.7) |
|  | Age 50-64 | 1.3 | (0.5-3.3) | 0.3 | (0.1-1.2) | 3.6 | (1.0-13.5) | 1.0 | - | 1.0 | - | 0.9 | (0.1-6.4) | 2.1 | (0.1-40.2) | 0.6 | (0.2-1.8) |
|  | Age ≥65 | 1.0 | - | 1.0 | - | 1.0 | - | 1.0 | - | 1.0 | - | 1.0 | - | 1.0 | - | 1.0 | - |
|  | χ^2^_2-3_ (p-value) | 6.6 (0.086) | | 5.2 (0.158) | | 5.4 (0.145) | | 0.4 (0.823) | | 6.3* (0.042) | | 0.5 (0.921) | | 0.9 (0.831) | | 2.2 (0.540) | |
| Education |  |  |  |  |  |  |  |  |  |  |  |  |  |  |  |  |  |
|  | Low | 0.6 | (0.3-1.0) | 0.2* | (0.1-0.7) | 1.9 | (0.6-6.5) | 0.6 | (0.2-2.1) | 0.5 | (0.1-5.0) | 0.6 | (0.2-2.2) | 3.5 | (0.6-21.0) | 3.3* | (1.4-7.7) |
|  | Low average | 0.7 | (0.4-1.2) | 0.4 | (0.1-1.8) | 1.2 | (0.4-3.6) | 0.7 | (0.2-2.5) | 2.0 | (0.4-9.4) | 0.5 | (0.1-2.0) | - | - | 2.5* | (1.2-4.9) |
|  | High average | 0.8 | (0.5-1.3) | 0.2* | (0.1-0.7) | 1.6 | (0.6-4.1) | 0.9 | (0.2-3.9) | 0.8 | (0.2-2.8) | 1.0 | (0.3-3.1) | 2.2 | (0.8-6.0) | 1.7 | (0.9-3.0) |
|  | High | 1.0 | - | 1.0 | - | 1.0 | - | 1.0 | - | 1.0 | - | 1.0 | - | 1.0 | - | 1.0 | - |
|  | χ^2^_2-3_ (p-value) | 3.7 (0.292) | | 11.0* (0.012) | | 2.3 (0.519) | | 0.8 (0.853) | | 2.6 (0.466) | | 1.4 (0.708) | | 3.0 (0.224) | | 9.5* (0.024) | |
| Marital status |  |  |  |  |  |  |  |  |  |  |  |  |  |  |  |  |  |
|  | Married-cohabitating | 1.0 | - | 1.0 | - | 1.0 | - | 1.0 | - | 1.0 | - | 1.0 | - | 1.0 | - | 1.0 | - |
|  | Previously married | 1.2 | (0.8-1.9) | 1.3 | (0.4-5.0) | 0.7 | (0.3-1.8) | 0.7 | (0.3-2.1) | 2.0 | (0.5-7.5) | 0.7 | (0.2-2.0) | 1.0 | (0.3-3.7) | 1.0 | (0.6-1.8) |
|  | Never married | 1.1 | (0.7-1.7) | 1.3 | (0.5-3.9) | 0.6 | (0.2-1.5) | 0.9 | (0.4-2.2) | 0.8 | (0.3-2.4) | 2.3 | (0.8-6.8) | 0.6 | (0.2-1.6) | 1.2 | (0.7-2.0) |
|  | χ^2^_2_ (p-value) | 0.7 (0.720) | | 0.3 (0.851) | | 1.2 (0.537) | | 0.4 (0.834) | | 1.4 (0.501) | | 4.9 (0.088) | | 1.2 (0.552) | | 0.5 (0.794) | |
| Income |  |  |  |  |  |  |  |  |  |  |  |  |  |  |  |  |  |
|  | Low | 0.8 | (0.5-1.3) | 1.2 | (0.3-4.4) | 1.6 | (0.7-3.9) | 1.2 | (0.3-4.5) | 1.3 | (0.4-4.5) | 0.8 | (0.2-2.4) | 0.5 | (0.2-1.7) | 0.8 | (0.4-1.6) |
|  | Low average | 1.1 | (0.7-1.8) | 2.4 | (0.8-7.3) | 1.0 | (0.4-2.4) | 0.7 | (0.2-2.5) | 1.8 | (0.4-7.2) | 0.7 | (0.3-2.0) | 0.7 | (0.1-3.5) | 0.7 | (0.3-1.3) |
|  | High average | 0.9 | (0.5-1.6) | 1.8 | (0.6-5.3) | 1.3 | (0.4-4.6) | 0.4 | (0.1-1.7) | 2.8 | (0.4-18.1) | 0.9 | (0.3-3.2) | 0.5 | (0.1-2.4) | 0.8 | (0.4-1.6) |
|  | High | 1.0 | - | 1.0 | - | 1.0 | - | 1.0 | - | 1.0 | - | 1.0 | - | 1.0 | - | 1.0 | - |
|  | χ^2^_3_ (p-value) | 1.2 (0.755) | | 4.6 (0.200) | | 1.6 (0.660) | | 3.0 (0.396) | | 1.4 (0.695) | | 0.5 (0.920) | | 1.3 (0.739) | | 1.4 (0.713) | |
| *^a^Data are given as adjusted odds ratios (95% confidence interval) unless otherwise indicated* | | | | | | | | | | | | | | | | | |
| **Significant at p = .05, 2-sided test* | | | | | | | | | | | | | | | | | |
| *Reference categories are denoted as 1.0 -; Age groups 50-64 and 65+ were collapsed for Colombia and Medellin, Colombia. The Low Average category for Peru was excluded due to cells with zero-count.* | | | | | | | | | | | | | | | | | |
| *The degrees of freedom for each chi-square test is based upon the number of groups available in each main category* | | | | | | | | | | | | | | | | | |
| *Note: each row shows a separate logistic regression model with 12-month service use as the outcome variable, controlling for the other predictor variables (rows), survey, and all predictor-by-survey interaction dummies. The second column shows the overall adjusted predictor variable effect; the survey columns show to what extent the survey-specific adjusted predictor variable effect deviates from the overall adjusted predictor variable effect. For example, the survey-specific effect for females (versus males) in the U.S. can be obtained by multiplying the aOR = 1.2 (the overall effect) by the aOR = 1.0 (the country-specific deviation), i.e., aOR = 1.2* | | | | | | | | | | | | | | | | | |
| *Models include controls for groups of 12-Month DSM-IV / WMH CIDI disorders (any anxiety, any mood, any substance and any externalized)* | | | | | | | | | | | | | | | | | |
| *Intermittent explosive disorder was not assessed in Mexico and Medellin, were coded as zero; Imputed variables for alcohol and drug dependence were used for Colombia, Mexico, Peru and the U.S.; Lifetime ADHD was used in all countries, and was coded as zero for those with age > 45 in Colombia, Mexico, Peru and the U.S.* | | | | | | | | | | | | | | | | | |
